# Dr. J. W. Kasbeer

**Published:** 1894-09

**Authors:** 


### CORRESPONDENCE.

The following letter will in the main explain itself. It may be said, however, before giving it, that it is a matter of great gratification that this work has been taken up in so systematic a manner as has been done by Dr. J. W. Kasbeer.

Every institution in the country where it is found necessary to have a physician attached, should also have a good dentist as well. A well equipped and faithful dentist who should give all needed attention to the inmates of such institutions would render the labor of the physician much less than it ordinarily is. Not only soldiers' orphans but the orphans of every other class that have been brought into similar institutions for care and needed attention, should have some one to give special attention to the organs of mastication and the oral cavity as a whole.

Such service rendered these institutions should receive a liberal compensation from the public. In these institutions, usually there are children from infancy to fifteen years of age or over, which is the time of life when most scrupulous care should be given to the teeth. But to the letter:

"NORMAL, ILL.,

To the Editor of the Dental Register.

Dear Doctor:—

I wrote you sometime in November last asking a few questions in regard to my assuming a position in the Soldiers Orphan Home of this place. Finally I have made arrangements to assume the position of Dental Surgeon in the Institution, and will begin work in about a week or ten days.

The children there are from infants to fourteen years of age. The plan of procedure I have outlined is as follows:

1st. I have prepared a diagram of the teeth, both permanent and temporary, for the reception of such markings as I may see fit to place upon them. With this diagram I have prepared a blank about as follows:

Examination Blank,

Soldiers Orphan Home, Normal, Ill.

Date..........................

Name of patient..................................

..............................................

Color.................................. Complexion.. .......... .........

Age.............. ................ Sex.......... .....................

Color of Hair..................... Color of Eyes............ .. .....

Temperament..........................................................................

| Date. | Record of Operations. |
|---|---|
| | ................ ........ ....... ............ ..... .............. |
| | .................................. ................................ |
| | ................ ......................... ....................... |
| | ................ ................... . ......................... |
| | ................... ........................................ |
| | Remarks. |

Make and record thorough examination of all the mouths in the institution, using the blanks, one for each patient.

2d. Perform the most important or urgent work first, so arranging my visits to the patients as to make a complete round of the whole in at least one year.

3d. One year from the beginning compare the conditions of the mouth with the blanks of the first examination, doing such work as may then be necessary with the marks upon the respective blanks.

4th. Materials to be used: cement, Hill's stopping, gutta-percha, tin, etc., etc., and the character of other operations.

5th. Collect the children in the assembly room, and give them instructions as often as may be necessary as to the care of their teeth; the relation of their teeth to health, etc.

I shall be glad to have your suggestions in regard to regulating the teeth so early as fourteen years of age. I am,

Yours very truly,

J. W. KASBEER."

